# Genome-wide characterization of NBS-encoding genes in *Luffa cylindrica* and their putative roles in disease resistance

**DOI:** 10.3389/fgene.2026.1784002

**Published:** 2026-04-07

**Authors:** Xiaolin Yang, Fanke Zeng, Boyi Liu, Ziyan Yang, Hongyun Wei, Jian Gao, Peilin Wang, Xinqiong Liu

**Affiliations:** 1 Institute of Plant Protection and Soil Fertilizer, Hubei Academy of Agricultural Sciences, Key Laboratory for Sustainable Control of Crop Disease, Insect Pests and Weeds in Hubei, Wuhan, China; 2 Hubei Hongshan Laboratory, Wuhan, China; 3 College of Life Science, South-Central Minzu University, Wuhan, China; 4 Department of Plant Pathology, Southwest University, Chongqing, China; 5 Management Center of Yintiaoling National Nature Reserve, Chongqing, China

**Keywords:** disease resistance, gene expression, genome-wide characterization, *Luffa cylindrica*, NBS-LRR gene family, synteny analysis

## Abstract

Sponge gourd (*L. cylindrica* Mill.), a crucial dual-purpose crop in the Cucurbitaceae family, faces severe yield losses due to diseases like Fusarium wilt and Tomato leaf curl New Delhi virus (ToLCNDV) infection. The NBS-LRR (Nucleotide-Binding Site–Leucine-Rich Repeat) gene family, core components of plant Effector-Triggered Immunity (ETI), plays a vital role in pathogen defense. This study conducted a comprehensive genome-wide analysis of the NBS-LRR gene family in *Luffa cylindrica* using bioinformatics tools and transcriptome data. A total of 89 NBS-LRR genes were identified and classified into seven subfamilies: TIR(Toll/Interleukin-1 Receptor)-NBS-LRR(TNL) (15), TIR-NBS(TN)(16), CC (Coiled-Coil))-NBS-LRR(CNL)(8), CC-NBS (CN) (14), NBS(N)(23), NBS-LRR (NL) (10), and RN(RPW8-NBS) (3). These genes showed irregular distribution across 12 chromosomes, with the highest density on Chr08. Phylogenetic analysis revealed five primary clades, reflecting evolutionary relationships among subfamilies. Conserved domain and motif analysis indicated intra-subfamily conservation and inter-subfamily divergence, with all members containing the core NB-ARC domain. Promoter *cis*-acting element analysis identified 65 elements, with light-responsive and hormone/defense-stress-responsive elements being predominant, suggesting involvement in multiple biological processes. Intra-species synteny analysis found two homologous gene pairs between Chr02 and Chr06, while inter-species analysis showed closer evolutionary ties with cucumber (18 orthologous pairs) than *Arabidopsis* (1 ortholog) and no orthologs with rice. Tissue-specific expression analysis revealed highest expression in roots, and disease response analysis identified six genes associated with ToLCNDV resistance and nine genes linked to Fusarium wilt resistance. These findings provide valuable resources for understanding the molecular basis of disease resistance in *L. cylindrica* and accelerating disease-resistant breeding.

## Introduction

Sponge gourd (*Luffa cylindrica* Mill.), an annual climbing vine of the Cucurbitaceae family, holds significant economic value as a dual-purpose crop, with applications in food and medicine. Its various tissues, including fruits, leaves, loofah sponges, vines, seeds, and roots, exhibit medicinal properties such as anti-inflammatory, antioxidant, immunomodulatory, and anticancer activities (Zhao et al., 2022). Loofah sponge, derived from dried mature fruits and rich in cellulose and lignin, is widely used in medical dressings, eco-friendly cleaning products, and healthcare pillow fillings (Lin et al., 2025; Reyes-Perez et al., 2025). However, large-scale cultivation of sponge gourd is severely threatened by diseases, particularly Fusarium wilt caused by *Fusarium oxysporum* f. sp. *Luffae* and viral infections like ToLCNDV. These pathogens can cause 30%–50% yield losses and reduce fruit marketability, posing a major bottleneck to the sustainable development of the sponge gourd industry (Bindal et al., 2023; Krishnan et al., 2023).

Plants have evolved a two-tiered immune system to counteract pathogen invasion (Jones and Dangl, 2006). The first tier, PAMP-Triggered Immunity (PTI), is activated when cell surface-localized receptors recognize conserved pathogen-associated molecular patterns (PAMPs). The second tier, Effector-Triggered Immunity (ETI), relies on intracellular resistance (*R*) proteins that specifically recognize pathogen effectors, inducing robust defense responses (Jones and Dangl, 2006). As core components of the ETI pathway, *R* genes not only recognize pathogen effectors but also regulate the expression of downstream defense-related genes, ultimately triggering plant disease resistance. Based on the structural differences of their encoded protein domains, plant *R* genes are classified into multiple families, including NBS-LRR, LRR-TM (Leucine-Rich Repeat–Transmembrane domain), STK (Serine/Threonine Kinase), RLK (Receptor-Like Kinase), and SA-CC (Signal Anchor–Coiled-Coil) (Couto and Zipfel, 2016; Hulbert et al., 2001). Among these, the NBS-LRR gene family is one of the largest and most critical *R* gene families in plants, widely distributed in eukaryotic genomes and playing a pivotal role in defending against diverse pathogens such as fungi, bacteria, and viruses (Jones et al., 2016; Shao et al., 2019).

With the rapid advancement of genome sequencing technologies and bioinformatics tools, genome-wide identification and functional characterization of the NBS-LRR gene family have been conducted in various plant species. For example, 327 NBS-LRR genes were identified in cassava (Lozano et al., 2015), 156 in tobacco (Zhu et al., 2025), 100 in Actinidia chinensis (Wang et al., 2020), and 252 in pepper (Liu et al., 2025). NBS-LRR family proteins are characterized by conserved structural domains: the N-terminus typically contains TIR (Toll/Interleukin-1 Receptor), CC (Coiled-Coil), or RPW8 (Resistance to Powdery Mildew 8) domains. The TIR and CC domains are involved in activating downstream signaling pathways, while the RPW8 domain can confer broad-spectrum disease resistance. The central region contains a highly conserved NBS (Nucleotide-Binding Site) domain (also known as NB-ARC) that binds ATP or GTP to provide energy for signal transduction. The C-terminal LRR (Leucine-Rich Repeat) domain is responsible for the specific recognition of pathogens by perceiving pathogen effectors (Liu et al., 2007). Based on protein domain architecture, NBS-LRR genes are classified into eight subfamilies: TNL (TIR-NBS-LRR), TN (TIR-NBS), CNL (CC-NBS-LRR), CN (CC-NBS), RNL (RPW8-NBS-LRR), RN (RPW8-NBS), NL (NBS-LRR), and N (NBS-ARC) (Wan et al., 2013).

Previous studies have confirmed the critical role of the NBS-LRR gene family in disease resistance in Cucurbitaceae crops (Brotman et al., 2002; Mandal et al., 2020). For instance, certain NBS-LRR members in watermelon are significantly associated with resistance to gummy stem blight caused by *Ascochyta citrulline* (Hassan et al., 2019). In cucumber, NBS-LRR genes are significantly upregulated in response to downy mildew (*Pseudoperonospora cubensis*) and powdery mildew (*Sphaerotheca fuliginea*) (Zhang et al., 2022). Additionally, functional members of the NBS-LRR gene family, such as *GmKR3* (which confers resistance to soybean mosaic virus in soybean) and *RPP4* (which mediates resistance to downy mildew in *Arabidopsis thaliana*), have been successfully deployed in crop breeding programs to enhance disease resistance (van der Biezen et al., 2002; Xun et al., 2019). However, current research on *L. cylindrica* has predominantly focused on cultivation practices and quality-oriented breeding, with the exploration and exploitation of its resistance gene resources remaining largely inadequate. Notably, a comprehensive genome-wide identification, classification, and functional characterization of the NBS-LRR gene family in *L. cylindrica* has not been reported to date. In this study, we performed genome-wide identification and systematic analysis of the NBS-LRR gene family in *L. cylindrica* using bioinformatics approaches, encompassing analyses of physicochemical properties, chromosomal localization, phylogenetic relationships, conserved domains and motifs, promoter *cis*-acting elements, and intra- and interspecific collinearity. Furthermore, we integrated transcriptome sequencing data to investigate the spatiotemporal expression patterns of NBS-LRR genes across diverse tissues and their transcriptional responses to ToLCNDV infection, as well as to compare the expression profiles between Fusarium wilt-resistant and susceptible varieties. Collectively, the findings of this study are anticipated to provide valuable insights for accelerating the development of disease-resistant *L. cylindrica* cultivars through molecular breeding strategies.

## Results

### Identification and classification of the NBS-LRR gene family in *Luffa cylindrica*

A total of 89 NBS-LRR family genes were identified in the *L. cylindrica* genome through a comprehensive strategy combining BLAST homology searches and HMM analysis. Conserved domain validation using SMART, NCBI CD-search, and InterPro, followed by manual curation, ensured all candidate sequences contained the core NBS domain. Based on distinct domain architectures, these 89 genes were classified into seven subfamilies: TNL (15 members), TN (16 members), CNL (8 members), CN (14 members), N (23 members), NL (10 members), and RN (3 members).

**FIGURE 1:**
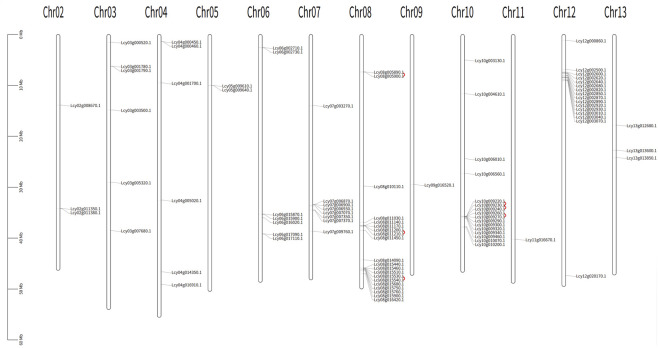
Chromosomal localization and tandem duplication genes of *Luffa* NBS-LRR genes. The map is obtained based on chromosomal coordinates of NBS-LRR genes from annotation files of the *Luffa cylindrical* genome (v3.0) using Mapchart. The scale on the far left of the image indicates the length of the chromosomes in megabases (Mb). Chromosome numbers were labeled at the top of each chromosome. Tandem repeat genes were illustrated by red lines.

The 89 NBS-LRR genes were irregularly distributed across 12 chromosomes, with no genes mapped to chromosome 1 (Figure 1). Chromosome eight had the highest gene density, harboring 20 members, followed by chromosomes 10 and 12 (16 genes each). In contrast, chromosomes 9 and 11 contained the fewest NBS-LRR genes, with only one gene per chromosome. Tandem repeat genes were illustrated by red lines in Figure 1, and a total of 12 tandem repeat sequences were identified on chromosomes 8 and 11.

### Physicochemical properties and subcellular localization

Significant variation was observed in the physicochemical properties of *L. cylindrica* NBS-LRR proteins. The amino acid sequence length ranged from 130 aa (*Lcy04g000460.1*) to 1,664 aa (*Lcy12g002620.1*), with corresponding predicted molecular weights of 14.7 kDa (*Lcy04g000460.1*) to 189.7 kDa (*Lcy12g002620.1*). The theoretical isoelectric point (pI) values varied from 5.24 to 9.08. Regarding protein stability, 25 proteins had an instability index <40 (relatively stable), while the remaining 64 were predicted to be unstable (instability index ≥40). For the aliphatic index, 45 proteins had a value <100 (more hydrophilic) and 44 had a value >100 (more hydrophobic) (Supplementary Table S1).

Subcellular localization prediction using the WoLF PSORT online tool showed that *L. cylindrica* NBS-LRR proteins were predominantly localized to the nucleus (42 members) and cytoplasm (34 members). A small proportion of proteins were predicted to target other compartments, including chloroplasts, peroxisomes, and mitochondria.

### Phylogenetic analysis of the NBS-LRR gene family

**FIGURE 2:**
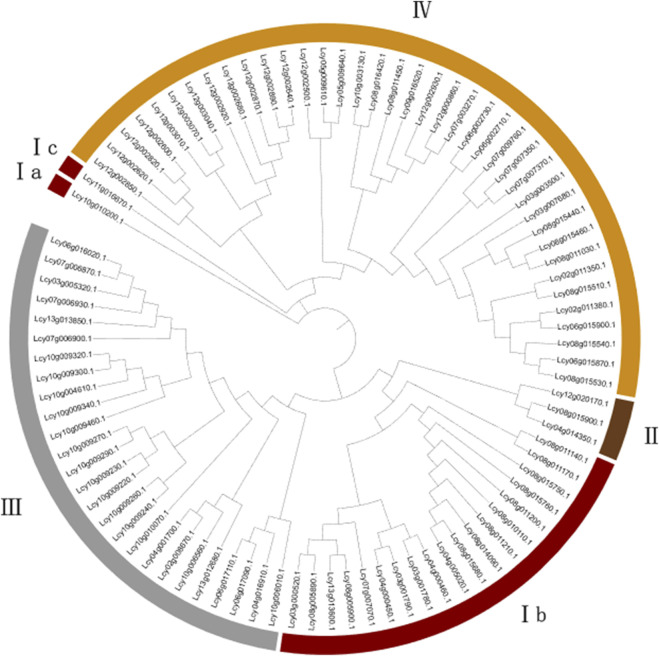
Phylogenetic tree of the NBS-LRR gene family in Luffa. The tree was constructed using IQ-TREE with 1,000 bootstrap replicates, and visualized using iTOL v7. Different clades represent distinct subfamilies.

**FIGURE 3:**
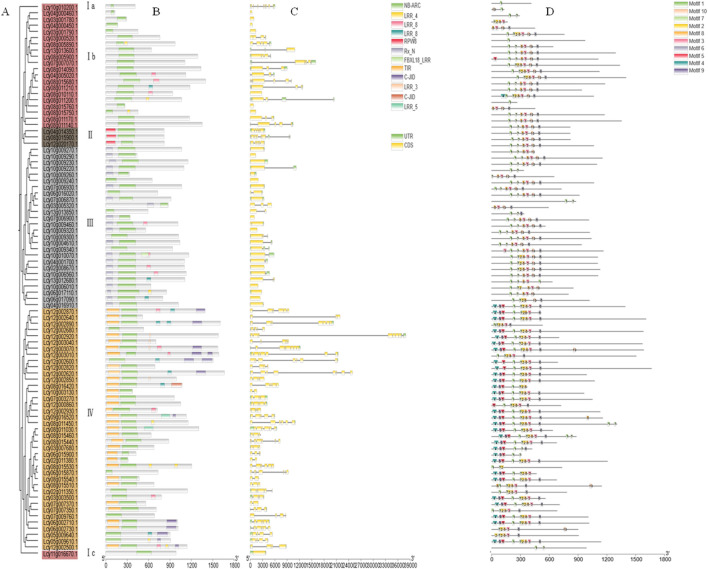
Phylogenetic and structure analysis of Luffa NBS-LRR gene family. **(A)** Phylogenetic tree of Luffa NBS-LRR gene family. The tree was constructed based on amino acid sequences using the Neighbor-Joining method with 1000 bootstrap replicates. **(B)** Conserved domain architectures of NBS-LRR proteins, visualized using TBtools. **(C)** Gene structures of Luffa NBS-LRR gene family. Gene structures were drawn based on genome annotation files. **(D)** Distribution of conserved motifs predicted by MEME Suite, visualized using TBtools. Different colors represent distinct motifs.

A phylogenetic tree was constructed based on the protein sequences of the 89 identified NBS-LRR family members (Figure 2 and Figure 3A). The tree was divided into five primary clades, including three major branches and two minor branches. Combined with gene structure, protein domain, and conserved motif analyses, the phylogenetic clustering facilitated further subfamily classification. Specifically, proteins *Lcy10g010200.1* and *Lcy11g016670.1*, which clustered in minor clades Ia and Ic, respectively, were classified into the N subfamily, with clade Ib also belonging to this subfamily. Major clade II contained members of the RN subfamily. Major clade III was predominantly composed of CNL and CN subfamily members, while major clade IV mainly included TN and TNL subfamily proteins.

### Conserved domains and motifs analysis

All 89 *L. cylindrica* NBS-LRR gene family members carry an NB-ARC domain (Figure 3B). The RPW8, Rx_N, and TIR domains are unique to subgroups II, III, and IV, respectively. LRR domains exist in all subgroups except II; their copy number varies among members but is consistently positioned at the 3′terminus.

Most *L. cylindrica* NBS-LRR genes have long CDS regions; CNL subfamily members, though few, possess notably extended CDS regions to support the synthesis of complex structural proteins. A minority of genes have short CDS regions, and except for the RN subfamily, these genes usually lack LRR or N-terminal domains. The TNL subfamily contains many genes with short CDS regions, a feature that may optimize rapid signal transduction in disease resistance. The gene family has 1–8 exons and 1–7 introns; subgroups II, III, and IV show slight differences in exon-intron number and distribution (suggesting conserved structures), while significant variations exist between these subgroups (Figure 3C).

Figure 3D shows that the number of conserved motifs in *L. cylindrica* NBS-LRR protein sequences varies substantially, ranging from 1 to 11, with each motif spanning 15 to 41 amino acids. Motif distribution analysis revealed that protein sequences within the same subgroup typically share similar motif compositions, whereas those across different subgroups exhibit distinct profiles. The vast majority of sequences feature a relatively conserved motif arrangement: motif 1–motif 7–motif 8–motif 3–motif 6. Specifically, sequences in subgroups I, II, and III predominantly adopt either the motif 1–motif 7–motif 8–motif 3–motif 10–motif 6 or motif 1–motif 2–motif 7–motif 8–motif 3–motif 10–motif 6 configuration, with a small subset lacking motif 6. In contrast, the conserved motifs in subgroup IV are almost exclusively organized as motif 2–motif 9–motif 1–motif 4–motif 6. Notably, only members of subgroup IV harbor the unique 5′terminal motif arrangement (motif 4–motif 9–motif 5), which constitutes the TIR domain. Variations in the copy number of identical motifs were also observed: within subgroup IV, *Lcy08g015440.1* and *Lcy08g011030.1* each contain two copies of the motif 1–motif 7 unit, whereas all other sequences in this subgroup possess only one copy.

### Promoter *cis*-acting elements analysis

**FIGURE 4:**
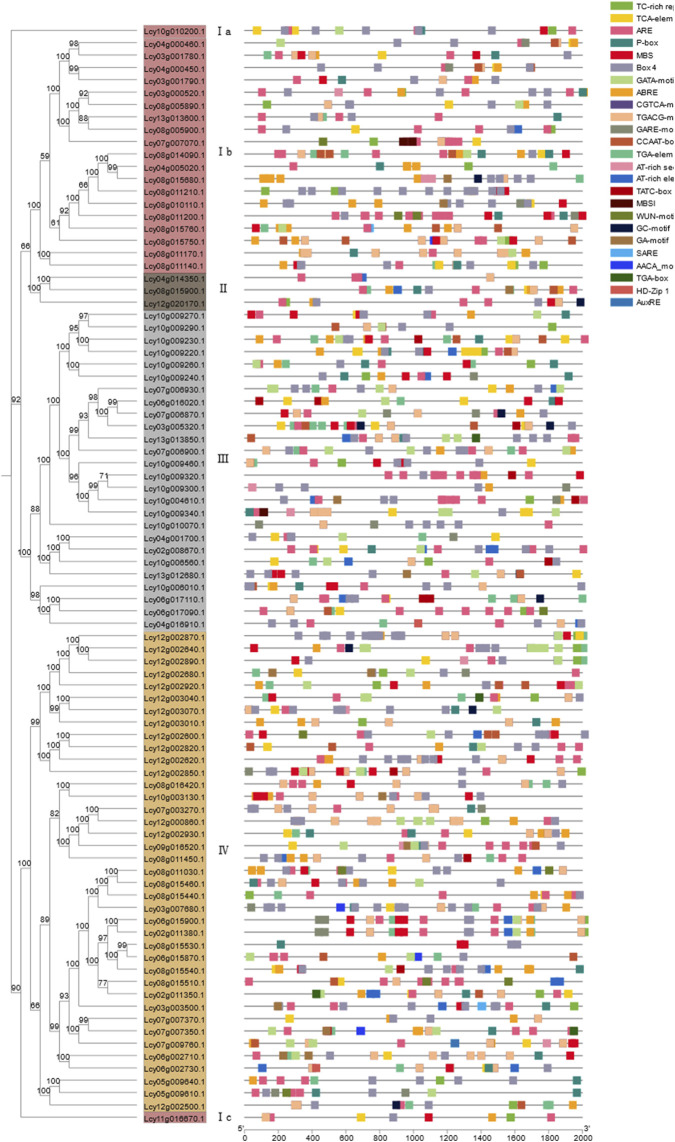
*Cis*-acting elements in the *Luffa* NBS-LRR gene family. The 2000 bp upstream sequences of NBS-LRR genes were analyzed using PlantCARE, and the distribution of *cis*-acting elements was visualized using TBtools. Different colors represent distinct types of *cis*-acting elements.

**FIGURE 5:**
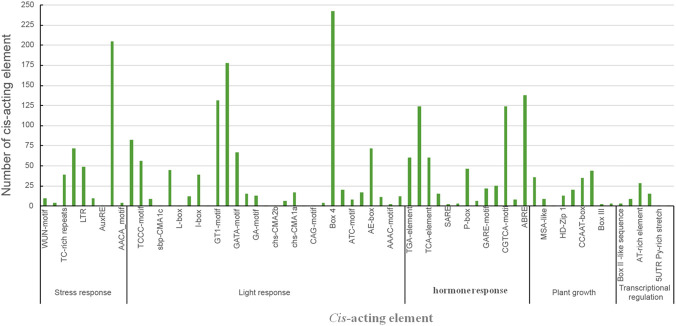
Statistics of *cis*-acting elements. The histogram shows the number of different categories of *cis*-acting elements, including transcriptional regulation, stress response, light response, and plant growth-related elements.

A systematic analysis of the 2000 bp promoter sequences upstream of *L. cylindrica* NBS-LRR genes identified 65 distinct *cis*-acting elements, classified into five major categories based on biological functions (Figures 4, 5). The first category includes defense and stress-responsive elements, such as LTR (low-temperature responsiveness), ARE (anaerobic induction), and WUN-motif (wound responsiveness). The second category consists of hormone-responsive elements, represented by TGACG-motif (methyl jasmonate responsiveness), TCA-element (salicylic acid responsiveness), and TATC-box (gibberellin responsiveness). The third category comprises elements related to plant growth, development, and cell cycle regulation, such as MSA-like, CAT-box, and GCN4_motif. The fourth category includes transcription-related cis-regulatory elements, e.g., 5′UTR Py-rich stretch, AT-rich element, and A-box. The fifth category is composed of light-responsive elements, including ACE, G-box, and GT1-motif.

Statistical analysis (Figure 5) showed that hormone-responsive and defense/stress-responsive elements together account for approximately 45% of the total cis-acting elements, while light-responsive elements alone represent 46%. This comprehensive characterization of cis-acting elements suggests that the *L. cylindrica* NBS-LRR gene family is likely involved in regulating multiple biological processes, including light signal transduction, hormone signaling pathways, and plant defense responses to abiotic and biotic stresses.

### Intra- and inter-species synteny analysis

**FIGURE 6:**
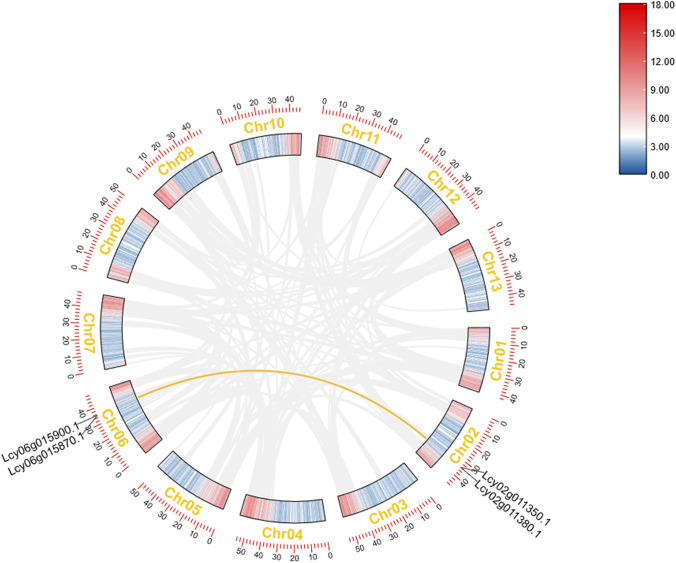
Intra-species synteny analysis of the *Luffa* NBS-LRR gene family. Collinear regions between chromosomes are indicated by lines, with homologous gene pairs labeled.

**FIGURE 7:**
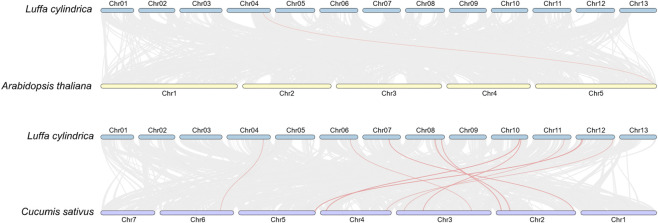
Inter-species synteny analysis of the *Luffa* NBS-LRR gene family. Syntenic relationships between *Luffa* and *Arabidopsis thaliana*, *Cucumis sativus*, and *Oryza sativa* are shown. Lines indicate orthologous gene pairs.

Intraspecific synteny analysis of *L*. *cylindrica* identified two pairs of homologous genes, all of which mapped to the collinear blocks between chromosome two and chromosome 6 (Figure 6). For interspecific synteny analysis (Figure 7), pairwise comparisons were performed between *L. cylindrica* and three representative species, namely, *A. thaliana* (eudicot), *Cucumis sativus* (cucumber, eudicot), and *O. sativa* (rice, monocot). Among the 89 NBS-LRR genes identified in *L. cylindrica*, only a single ortholog (*Lcy04g014350.1*) was conserved relative to *A. thaliana*. In contrast, 18 orthologous gene pairs were detected between *L. cylindrica* and *C. sativus*, involving the following *L. cylindrica* genes: *Lcy11g016670.1*, *Lcy10g010070.1*, *Lcy10g010200.1*, *Lcy10g009220.1*, *Lcy10g009300.1*, *Lcy10g009460.1*, *Lcy12g020170.1*, *Lcy12g002640.1*, *Lcy12g003010.1*, *Lcy12g002620.1*, *Lcy04g014350.1*, *Lcy06g017090.1*, *Lcy08g011140.1*, *Lcy08g011450.1*, *Lcy08g015680.1*, *Lcy08g015900.1*, *Lcy07g006870.1*, and *Lcy07g007070.1*. No orthologous relationships were found between the NBS-LRR genes of *L. cylindrica* and those of *Oryza sativa*.

### Ka/Ks analysis of two NBS-LRR paralogous pairs in *Luffa cylindrica*

To investigate the evolutionary selection pressure acting on members of the NBS-LRR gene family in *L. cylindrica*, we calculated the non-synonymous substitution rate (Ka) and synonymous substitution rate (Ks), as well as the Ka/Ks ratio, for two pairs of paralogous genes based on the results of intra-specific collinearity analysis within *L. cylindrica* (Supplementary Table S2). For the gene pair *Lcy06g015900.1* and *Lcy02g011380.1*, the Ka value was 0.02, the Ks value was 0.03, and the Ka/Ks ratio was 0.76. In contrast, the gene pair *Lcy06g015870.1* and *Lcy02g011350.1* exhibited significantly higher substitution rates, with a Ka value of 0.26, a Ks value of 0.38, and a Ka/Ks ratio of 0.68. Generally, a Ka/Ks ratio less than one indicates purifying selection, while a ratio greater than one indicates positive selection. The Ka/Ks ratios for both pairs of homologous genes were less than 1, indicating that both gene pairs have evolved under purifying selection.

### Tissue-specific expression patterns of NBS-LRR genes

**FIGURE 8:**
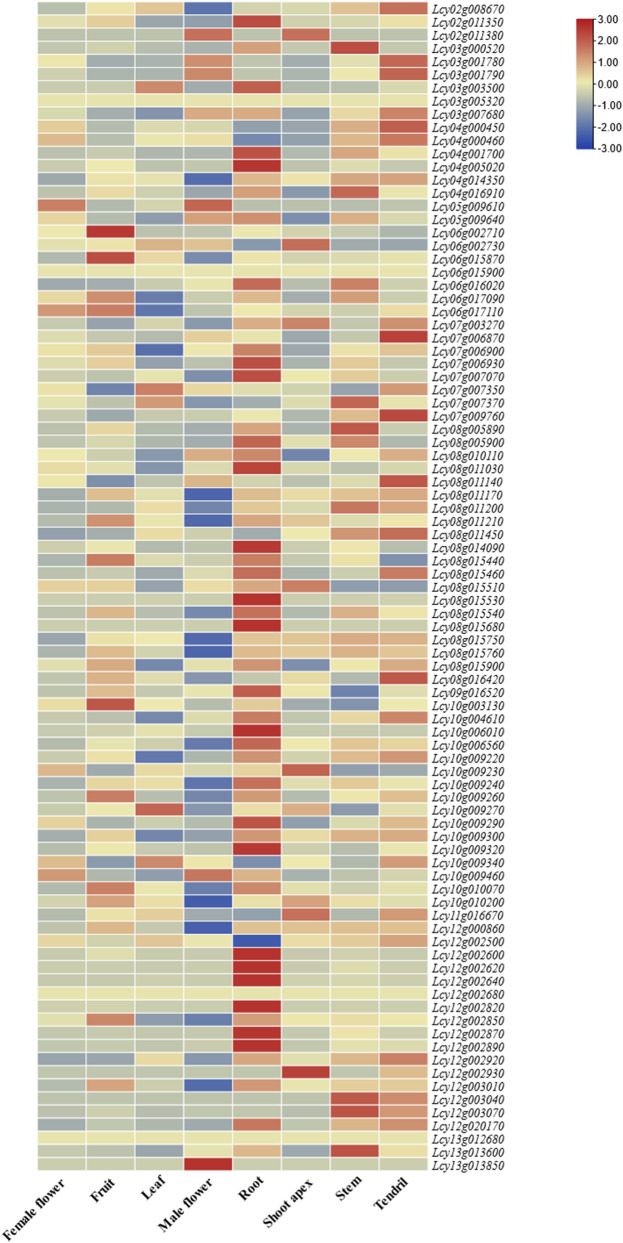
Expression patterns of the *Luffa* NBS-LRR gene family in different tissues. The heatmap was generated based on FPKM values from RNA-seq data, showing expression levels in leaf, root, stem, tendril, female flower, fruit, male flower, and shoot apex. Color intensity represents relative expression levels.

RNA-seq datasets derived from seven distinct organs of *L*. *cylindrica* were employed to characterize the tissue-specific expression profiles of NBS-LRR genes (Figure 8). Genes with an average FPKM value <1 across all samples were excluded from subsequent analyses, and the remaining 85 genes were used to construct a gene expression heatmap. Tissue-specific expression profiling revealed that 85 of the 89 identified NBS-LRR genes were ubiquitously expressed across all tested tissues, whereas four genes (*Lcy03g005320.1*, *Lcy06g015900.1*, *Lcy12g002680.1*, and *Lcy13g012680.1*) showed no detectable expression in any of the examined organs.

Hierarchical clustering analysis categorized the expression patterns of these 85 expressed genes into three distinct classes: low-level constitutive expression, tissue-specific expression, and widespread constitutive expression. As illustrated in Figure 8, the NBS-LRR gene family exhibited the highest expression levels in roots, a pattern presumably associated with the heightened requirement for defense against soil-borne pathogens. In contrast, these genes maintained basal expression levels in flowers, fruits, stems, and leaves, indicating their involvement in constitutive defense responses that underpin normal plant growth and basal disease resistance. The differential expression of individual NBS-LRR genes across diverse tissues, coupled with the variable expression abundances of distinct family members within the same tissue, underscores the complexity of NBS-LRR-mediated disease resistance regulatory networks and suggests the occurrence of tissue-specific functional divergence among its members.

### Expression analysis of NBS-LRR genes in response to diseases

#### Expression response to ToLCNDV infection

**FIGURE 9:**
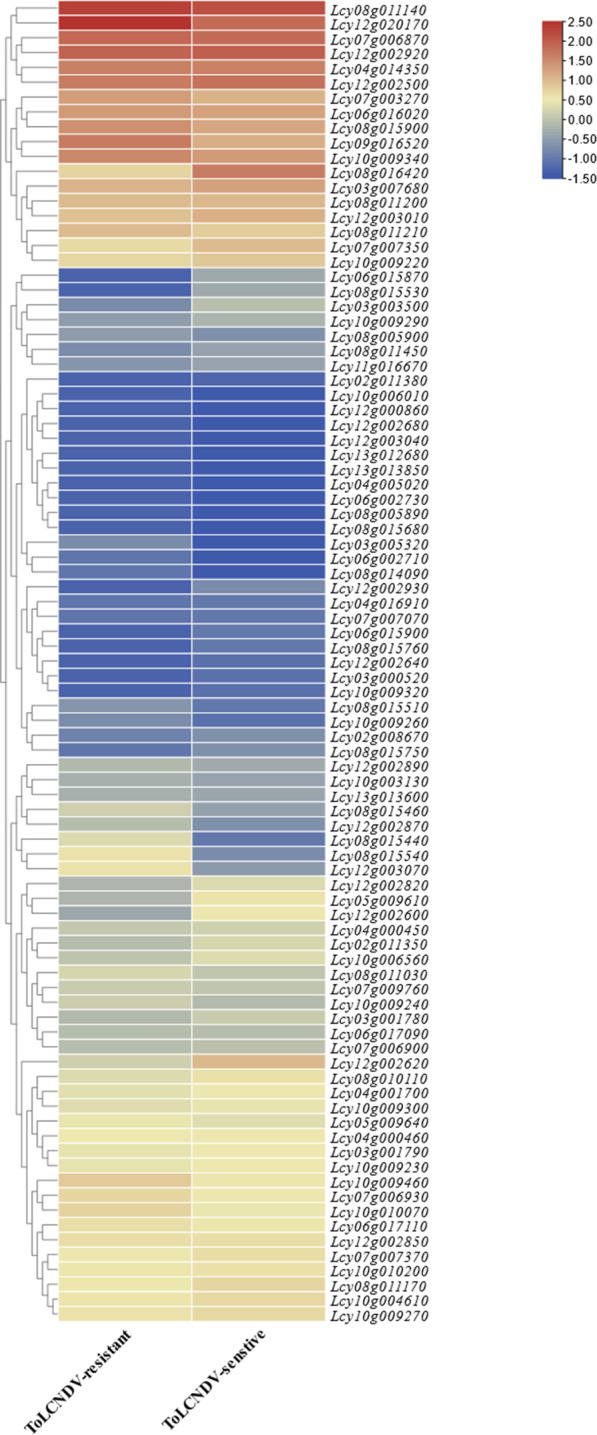
Expression patterns of the *Luffa* NBS-LRR gene family in leaves of ToLCNDV-resistant and susceptible varieties. The heatmap was generated based on FPKM values from RNA-seq data, with color intensity indicating relative expression levels.

RNA-seq datasets from ToLCNDV-resistant and susceptible *L. cylindrica* varieties were used to analyze the expression patterns of NBS-LRR genes in leaves (Figure 9). The results showed that NBS-LRR genes generally exhibited significantly higher expression levels in resistant varieties compared to susceptible ones, indicating that the NBS-LRR gene family is transcriptionally upregulated in response to ToLCNDV infection, thereby contributing to enhanced disease resistance. Notably, NBS-LRR genes maintained a certain level of basal expression in both resistant and susceptible varieties, suggesting they belong to constitutively expressed gene families and may serve as the molecular basis for basal defense responses. Among all tested genes, six specific members—*Lcy03g005320.1*, *Lcy08g015440.1*, *Lcy08g015460.1*, *Lcy08g015540.1*, *Lcy12g002870.1*, and *Lcy12g003070.1*—showed remarkably higher expression levels in resistant varieties relative to susceptible ones, strongly implying that these six genes may act as key resistance-related genes mediating the specific defense response against ToLCNDV in *L. cylindrica*.

#### Expression response to fusarium wilt infection

**FIGURE 10:**
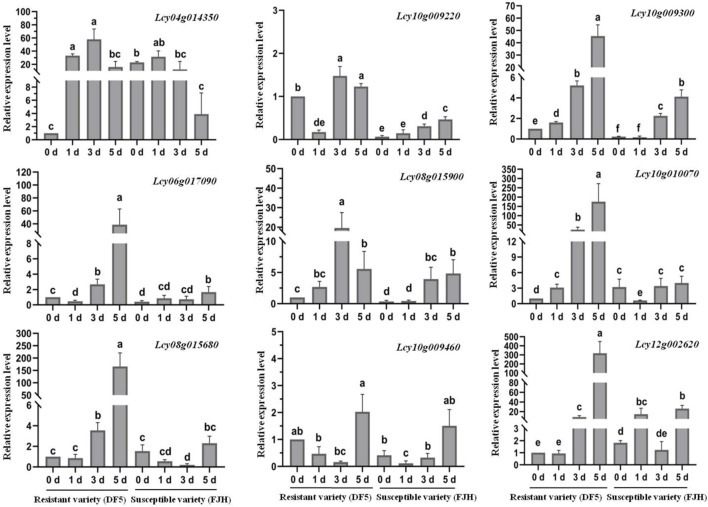
Expression analysis of NBS-LRR genes in resistant and susceptible varieties of *Luffa cylindrica* in response to fusarium wilt. Error bars represent ±SE of the means of three technical replicates. Different letters above the bars indicate significant differences.

Resistant and susceptible varieties of *L. cylindrica* were inoculated with Fusarium oxysporum, and qRT-PCR was employed to detect expression divergence of 18 NBS-LRR genes exhibiting high conservation between *L. cylindrica* and cucumber. The results demonstrated that the transcript levels of nine out of these 18 genes (*Lcy04g014350.1*, *Lcy06g017090.1*, *Lcy08g015680.1*, *Lcy08g015900.1*, *Lcy10g009220.1*, *Lcy10g009300.1*, *Lcy10g009460.1*, *Lcy10g010070.1*, *Lcy12g002620.1*) were significantly elevated in resistant varieties relative to susceptible counterparts at multiple time points post-inoculation (Figure 10). For instance, in the resistant cultivar ‘DF5’, *Lcy04g014350.1*, *Lcy10g009300.1*, and *Lcy10g010070.1* displayed marked upregulation at 1, 3, and 5 days post-inoculation (dpi), in contrast to the susceptible cultivar ‘FJH’. By comparison, *Lcy06g017090.1*, *Lcy08g015680.1*, *Lcy08g015900.1*, and *Lcy12g002620.1* were expressed at significantly higher levels in ‘DF5’ specifically at three and five dpi. The remaining nine genes showed no statistically significant expression differences between the two varietal groups.

These findings suggest that the aforementioned 9 NBS-LRR genes may function as key resistance-related determinants against *L. cylindrica* Fusarium wilt, thereby providing valuable insights for further elucidating the molecular mechanisms underlying Fusarium wilt resistance in *L. cylindrica*. Furthermore, the conservation of these genes between *L. cylindrica* and cucumber indicates evolutionary conservation of disease resistance pathways among cucurbit crops, laying a solid foundation for cross-species resistance gene mining and translational utilization in breeding programs.

## Discussion

The NBS-LRR gene family, a core component of plant innate immunity, has been the subject of extensive investigation across diverse plant taxa (Shao et al., 2019). For instance, 202 NBS-LRR genes have been annotated in *A*. *thaliana*, 545 in *O*. *sativa* (rice), and 411 in *Setaria italica* (foxtail millet), with over 100 functional NBS-LRR genes cloned and characterized from various plant species to date (Meyers et al., 2003; Zhao et al., 2016; Zhou et al., 2004). Liu et al. (2023) categorized NBS-LRR genes into three evolutionarily ancient subclasses—TNL, CNL, and RNL—noting that RNLs are highly conserved across lineages and primarily mediate downstream immune signal transduction. In contrast, TNLs and CNLs are specialized for pathogen recognition and exhibit lineage-specific expansion patterns (Shao et al., 2016). Functional divergence among these three subfamilies is hypothesized to drive differential evolutionary pressures, thereby shaping distinct adaptive trajectories and evolutionary pathways in response to pathogen challenges (Liu et al., 2025).

In the present study, a genome-wide screening pipeline identified 89 NBS-LRR genes in *L*. *cylindrica*, which were further classified into seven subfamilies based on conserved domain architectures. The relatively large number of members in the N (23 genes) and NL (10 genes) subfamilies suggests that these clades may fulfill critical roles in *L. cylindrica* immune responses or have undergone selective retention to maintain evolutionary conservation and functional diversity. The subcellular localization predictions targeting these proteins primarily to the nucleus and cytoplasm. During pathogen infection, NBS-LRR proteins are rapidly synthesized and activated to trigger immune cascades; upon activation, a subset translocates to the nucleus to interact with transcription factors and modulate defense gene expression. Such inherent instability is likely a functional trait, enabling dynamic and precise regulation of defense signaling through mechanisms such as rapid proteolytic degradation, conformational switching, or intracellular trafficking (Wang et al., 2023). The uneven distribution of NBS-LRR genes across 11 of the 12 *L. cylindrica* chromosomes (with no genes mapped to Chr01) is consistent with the random chromosomal distribution patterns reported in other plant species. Furthermore, 12 tandem repeat clusters were identified on Chr08 and Chr11, and two pairs of homologous NBS-LRR genes were localized to collinear regions between Chr02 and Chr06, indicating that tandem duplication and segmental duplication may have jointly contributed to the expansion of this gene family in *L. cylindrica*.

Length polymorphism of NBS-LRR genes is predominantly driven by variation in the copy number of LRR domains, a feature closely linked to the specificity of pathogen recognition (Bryan et al., 2000; Liu et al., 2025). For example, in *Panax ginseng*, CNL subfamily genes exhibit significantly higher expression levels than TNLs during the early stage of root rot infection (Ning et al., 2021). Shortened coding sequences (CDSs) of NBS-LRR genes often correlate with the loss of accessory domains (e.g., CC, TIR, or LRR), which may redirect the functional focus of these proteins to core NB-ARC domain-mediated signal transduction (Qi et al., 2012). Based on this precedent, we hypothesize that the long CDS lengths of most *L. cylindrica* NBS-LRR genes may encode proteins with elaborate domain architectures (e.g., multiple tandem LRRs), which enhance the specificity of pathogen effector recognition (DeYoung and Innes, 2006). Conversely, the small subset of *L. cylindrica* NBS-LRR genes with truncated CDSs may function primarily in immune signal propagation, facilitating rapid defense activation during pathogen invasion (Zhu et al., 2025). Comparative analysis of gene structures within the *L. cylindrica* NBS-LRR family revealed high conservation of intron-exon number and arrangement among members of the same subfamily, whereas substantial structural divergence was observed between different subfamilies. Collectively, the conserved domains, gene structures, and motif compositions within subfamilies, coupled with inter-subfamily variability, suggest that NBS-LRR genes within the same subfamily may exert analogous functions and respond to similar pathogen stimuli during immune responses.

*Cis*-acting regulatory elements (CAREs) play a pivotal role in modulating the spatiotemporal expression of NBS-LRR genes (Luo et al., 2025; Zhu et al., 2025). A recent study on the pepper (*Capsicum annuum*) NBS-LRR gene family demonstrated that light-responsive CAREs account for 38.7% of all identified elements, while hormone-responsive and defense/stress-responsive elements together constitute 50.7% of the total (Liu et al., 2025). This finding implies that light signaling pathways coordinate with defense responses in pepper to resolve the energy allocation trade-off between photosynthesis and pathogen resistance (Liu et al., 2025). In the current study, approximately 45% of CAREs in *L. cylindrica* NBS-LRR gene promoters are associated with hormone signaling and defense/stress responses, and light-responsive elements account for 46% of the total. These results indicate that the *L. cylindrica* NBS-LRR gene family is integrated into a complex regulatory network encompassing light signaling, hormone transduction, and stress responses, highlighting the multi-layered regulation of plant immune systems.

Interspecific synteny analysis provided insights into the evolutionary relationships of *L. cylindrica* NBS-LRR genes with those of other angiosperms. The Ka/Ks analysis of the two pairs of NBS-LRR paralogous genes in *L. cylindrica* revealed that both gene pairs have evolved under purifying selection. This finding is consistent with previous studies on plant disease resistance genes, where NBS-LRR genes are typically subject to strong purifying selection to maintain the critical structural domains essential for their resistance functions (Du Preez et al., 2008; Zhong et al., 2015). This purifying selection acting on the NBS-LRR genes in *L. cylindrica* likely preserves their original disease resistance functions, enabling their involvement in pathogen recognition and defense signaling (Morata and Puigdomènech, 2017). Notably, both pairs of homologous genes are annotated as TMV resistance protein N-like in the gene annotation files. The purifying selection may have maintained their characteristics as TMV resistance protein N-like (Dinesh-Kumar et al., 2000), further demonstrating the importance of this purifying selection in preserving the resistance functions of NBS-LRR genes in *L. cylindrica*. Furthermore, although both gene pairs are under purifying selection, they exhibit significantly different evolutionary rates (Zhong et al., 2022). The *Lcy06g015870.1-Lcy02g011350.1* gene pair shows notably higher Ka and Ks values, suggesting that it experienced an earlier divergence event and a longer independent evolutionary history.

Only one orthologous NBS-LRR gene pair was identified between *L. cylindrica* and *A. thaliana*, 18 conserved pairs were detected between *L. cylindrica* and cucumber (*C. sativus*), and no orthologs were found between *L. cylindrica* and the monocot *O. sativa*. These results align with the established angiosperm phylogenetic framework, indicating a relatively distant evolutionary relationship between *L. cylindrica* and monocots, a moderate affinity with the eudicot *A. thaliana*, and a close evolutionary kinship with cucumber—a fellow member of the Cucurbitaceae family. Notably, the gene *Lcy04g014350.1* possesses orthologs in both cucumber and *A. thaliana*, suggesting that it originated from a common ancestral locus and has retained conserved structural and functional features throughout eudicot evolution. Further investigation revealed that the *A. thaliana* orthologs of *Lcy04g014350.1* are *At5g66900* (NRG1.1) and *At5g04720* (ADR1-L2). *At5g66900* is dispensable for salicylic acid (SA) induction but is required for the execution of hypersensitive response (HR) cell death (Castel et al., 2019), while *At5g04720* acts as a key transducer of immune signals and participates in the SA-dependent defense pathway (Bonardi et al., 2011). These observations suggest that *Lcy04g014350.1* may play a conserved role in mediating HR and SA signaling during the immune response of *L. cylindrica*.

Roots represent the primary interface between plants and soil-borne pathogens, which can invade host tissues *via* spore germination, hyphal penetration, or direct physical disruption (Chuberre et al., 2018). Consistent with findings in apple (*Malus domestica*), where NBS-LRR genes exhibit peak expression levels in roots and stems (Arya et al., 2014), the *L. cylindrica* NBS-LRR gene family showed the highest transcript abundance in roots. This expression pattern is likely an adaptive trait that enhances defense against soil-borne pathogens such as *Fusarium oxysporum*, the causal agent of fusarium wilt. In plant-pathogen interactions, NBS-LRR genes typically exhibit elevated expression in resistant varieties compared to susceptible counterparts, while maintaining basal constitutive expression in both, a pattern observed in tomato (*Solanum lycopersicum*) in response to ToLCNDV infection (Kushwaha et al., 2015). In *L. cylindrica*, six NBS-LRR genes (*Lcy03g005320.1*, *Lcy08g015440.1*, *Lcy08g015460.1*, *Lcy08g015540.1*, *Lcy10g009260.1*, *Lcy12g002870.1*) were found to be significantly upregulated in ToLCNDV-resistant varieties, indicating their potential role in antiviral defense. For Fusarium wilt resistance, nine NBS-LRR genes (*Lcy04g014350.1*, *Lcy06g017090.1*, *Lcy08g015680.1*, *Lcy08g015900.1*, *Lcy10g009220.1*, *Lcy10g009300.1*, *Lcy10g009460.1*, *Lcy10g010070.1*, *Lcy12g002620.1*) showed markedly higher expression levels in resistant *L. cylindrica* varieties post-inoculation with *F. oxysporum*, identifying these genes as key candidate determinants of fusarium wilt resistance.

To our knowledge, this study represents the first comprehensive characterization of the NBS-LRR gene family in *L. cylindrica* and the first report of candidate resistance genes associated with Fusarium wilt in this crop. The findings provide a robust genetic framework for future functional validation of NBS-LRR genes and lay the foundation for molecular breeding strategies aimed at improving disease resistance in *L. cylindrica*.

## Materials and methods

### Identification of the NBS-LRR gene family in *Luffa cylindrica*

Protein sequences, genome sequences, and annotation data of *L. cylindrica* were downloaded from CuGenDBv2 (http://cucurbitgenomics.org/v2/) (Yu et al., 2023). NBS-LRR gene sequences of *A. thaliana* (reference dataset) were retrieved from the NIBLRRS platform (https://niblrrs.ucdavis.edu/). Preliminary candidate genes were identified *via* local BLAST (TBtools (Chen et al., 2020)) using *A. thaliana* NBS-LRR proteins as queries (*E*-value <1 × 10^−5^), followed by HMMER searches against the NBS domain model (Pfam: PF00931, *E*-value <1 × 10^−5^) (Liu et al., 2025). Conserved domain validation and false positive removal were conducted with SMART (Genomic mode), NCBI Batch CD-search (CDD database, *E*-value = 0.01), and InterPro. Sequences lacking a complete core NBS domain were manually excluded; *bona fide* NBS-LRR members were categorized into subfamilies according to conserved domain combinations (e.g., TIR, CC, RPW8, LRR).

### Physicochemical property analysis and subcellular localization prediction

The physicochemical properties (amino acid count, molecular weight, isoelectric point, instability index, and aliphatic index) of *L. cylindrica* NBS-LRR proteins were analyzed via the Protein Parameter Calc function in TBtools. Subcellular localization of these proteins was predicted using the plant-specific module of the online tool WoLF PSORT (https://wolfpsort.hgc.jp/).

### Chromosomal localization analysis

Based on *L. cylindrica* genome annotation data, chromosomal positions of the identified NBS-LRR genes were extracted. TBtools was utilized to analyze NBS-LRR gene density and detect tandem duplication events. A chromosomal localization map was constructed with MapChart, using chromosomal coordinates of NBS-LRR genes from the *L. cylindrica* genome (v3.0) annotation files; the leftmost scale indicates chromosome length in megabases (Mb), chromosome numbers are labeled at the top of each chromosome, and tandemly duplicated genes are marked with red lines.

### Phylogenetic analysis

Protein sequences of L. cylindrica NBS-LRR genes were aligned with MAFFT v7 (https://mafft.cbrc.jp/alignment/software/) using the auto-selected optimal algorithm. A phylogenetic tree was constructed in IQ-TREE with the best-fit substitution model determined automatically and 1,000 bootstrap replicates for branch support evaluation. The tree was annotated and visualized using the online tool iTOL v7 (https://itol.embl.de/) to generate a publication-quality figure.

### Conserved domain, motif, and gene structure analysis

Conserved domains of the identified NBS-LRR proteins were analyzed via NCBI Batch CD-search (https://www.ncbi.nlm.nih.gov/Structure/bwrpsb/bwrpsb.cgi) against the CDD database with an E-value threshold of 0.01, and their domain architectures were visualized using TBtools. Conserved protein motifs were predicted by the MEME Suite (https://meme-suite.org/meme/index.html) with the number of motifs set to 10 and other parameters at default values; motif distributions were also visualized using TBtools. Additionally, the gene structures (e.g., exons, introns) of NBS-LRR genes were extracted from genome annotation data and visualized via TBtools.

### Promoter *Cis*-acting element analysis

The 2000 bp upstream sequences from the start codon of NBS-LRR genes were extracted using TBtools. *Cis*-acting regulatory elements in these promoter sequences were predicted via the PlantCARE online tool (http://bioinformatics.psb.ugent.be/webtools/plantcare/html/). The criteria for selecting cis-regulatory elements are as follows: Only elements with a similarity score ≥90% compared to the database matrix were retained. Elements without functional annotations and those without clear names were excluded, and only elements relevant to this study were kept. All obtained elements were classified and counted according to their annotated functions. The distribution characteristics of the *cis*-acting elements and the results of the statistical analysis were visualized using TBtools software.

### Intra- and inter-species collinearity analysis

Genome data were retrieved from the following databases: *A*. *thaliana* (TAIR, https://www.arabidopsis.org/), *O. sativa* Japonica Group (Ensembl Plants, https://plants.ensembl.org/index.html), cucumber (CuGenDBv2, http://cucurbitgenomics.org/v2/), and *L*. *cylindrica* (CuGenDBv2). Intra-species collinearity of *L. cylindrica* NBS-LRR genes was analyzed using TBtools, with a collinearity diagram generated. For inter-species collinearity, pairwise syntenic analyses were performed between *L. cylindrica* and three representative species (*A. thaliana*, rice, and cucumber) via TBtools with default parameters, and a collinearity diagram was constructed to visualize inter-species syntenic relationships.

### Ka/Ks analysis of two NBS-LRR paralogous pairs in *Luffa cylindrica*

The CDS sequences of *L. cylindrica* were downloaded from the CuGenDBv2 database. The non-synonymous substitution rate (Ka) and synonymous substitution rate (Ks), as well as the Ka/Ks ratio, for two pairs of NBS-LRR paralogous genes in *L. cylindrica* were calculated using the Simple Ka/Ks Calculator tool (NG method) in TBtools.

### Tissue-specific expression analysis

RNA-seq datasets of *L*. *cylindrica* were retrieved from CuGenDBv2. Fragments Per Kilobase of transcript per Million fragments mapped (FPKM) values derived from Project PRJNA732226 were used to characterize the expression patterns of NBS-LRR genes across different tissues. Following FPKM data normalization, a heatmap was generated using TBtools to visualize the expression levels of NBS-LRR genes in leaf, root, stem, tendril, female flower, fruit, male flower, and shoot apex tissues.

### Expression analysis in response to ToLCNDV infection

FPKM data from Project PRJNA268703 were utilized to analyze the expression profiles of NBS-LRR genes in leaves of Tomato leaf curl New Delhi virus (ToLCNDV)-resistant and susceptible *L. cylindrica* varieties. After data normalization, a heatmap was constructed via TBtools to compare the expression differences of NBS-LRR genes between the two variety types.

### Quantitative real-time PCR (qRT-PCR) analysis for fusarium wilt resistance

Fourteen-day-old seedlings of the Fusarium wilt-resistant *L. cylindrica* variety ‘DF5′ and susceptible variety ‘FJH’ were inoculated with *Fusarium oxysporum*. The *F. oxysporum* isolate was cultured on potato dextrose agar (PDA) medium at 28 °C ± 1 °C in the dark for 1–2 weeks until the Petri dish was fully colonized by mycelia. The fungal spore concentration was adjusted to a final density of 1 × 10^7^ spores/mL. For inoculation, seedlings were carefully removed from pots without damaging the rhizomes; root tips were wounded with sterile scissors, and plants were immersed in the spore suspension for approximately 30 min. Control plants were immersed in sterile water. After inoculation, seedlings were replanted into pots and maintained in an artificial climate chamber under conditions of 28 °C ± 1 °C (light period), 26 °C ± 1 °C (dark period), and 60% relative humidity. Newly emerged leaves were sampled at 0, 1, 3, and 5 days post-inoculation (dpi), immediately frozen in liquid nitrogen, and stored at −80 °C for subsequent analysis.

Total RNA was extracted using Vezol reagent (Vazyme Bio, Nanjing, China). First-strand cDNA was synthesized with HiScript® II Q RT SuperMix for qPCR (+gDNA wiper) (Vazyme Bio, Nanjing, China). qRT-PCR was performed to analyze the expression patterns of nine NBS-encoding genes (*Lcy04g014350.1*, *Lcy06g017090.1*, *Lcy08g015680.1*, *Lcy08g015900.1*, *Lcy10g009220.1*, *Lcy10g009300.1*, *Lcy10g009460.1*, *Lcy10g010070.1*, *Lcy12g002620.1*) using QuanStudio™ Design & Analysis Software (Thermo Fisher Scientific Bio, MA, United States). Relative gene expression levels were calculated *via* the 2^^(−ΔΔCt)^ method, with *Lc18s rRNA* serving as the reference gene. Gene-specific primers were designed using SnapGene software (https://www.snapgene.com/) (Supplementary Table S3). The qRT-PCR experiments were performed with three technical and biological replicates, respectively. Statistical analyses, including analysis of variance (ANOVA) and significance testing, were performed using GraphPad Prism software (Dotmatics, Boston, MA, United States), with mean comparisons conducted via Tukey’s pairwise tests. Lv et al., 2015.

## Data Availability

The original contributions presented in the study are publicly available. This data can be found at the NCBI Sequence Read Archive (SRA) with the accession numbers PRJNA732226 and PRJNA268703.
