# Supplementary material for: Genome-wide characterization of NBS-encoding genes in Luffa cylindrica and their putative roles in disease resistance
Source: Front Genet. 2026 Apr 7;17:1784002. doi: 10.3389/fgene.2026.1784002 (PMC13095180; doi:10.3389/fgene.2026.1784002)
Supplement: Supplementary file 1 [file Table1.docx]

Table S1 **Physicochemical properties of the NBS-LRR gene family in *Luffa***

| Sequence ID | Number of amino acids | Molecular  Weight/ kDa | pI | Instability Index | Aliphatic Index |
| --- | --- | --- | --- | --- | --- |
| Lcy10g010200.1 | 412 | 45.1 | 6.09 | 52.75 | 91.55 |
| Lcy04g000460.1 | 130 | 14.66 | 8.97 | 37.35 | 83.23 |
| Lcy12g002870.1 | 1391 | 159.80 | 6.83 | 46.51 | 95.39 |
| Lcy04g014350.1 | 818 | 93.22 | 5.93 | 44.56 | 105.89 |
| Lcy08g016420.1 | 1072 | 122.29 | 8.02 | 44.79 | 89.94 |
| Lcy08g011030.1 | 1304 | 148.71 | 7.42 | 38.33 | 98.8 |
| Lcy07g003270.1 | 962 | 109.60 | 6.81 | 43.07 | 101.6 |
| Lcy12g002680.1 | 530 | 61.44 | 7.61 | 47.3 | 106.06 |
| Lcy03g001780.1 | 291 | 32.91 | 6.48 | 37.86 | 103.13 |
| Lcy03g007680.1 | 677 | 77.47 | 7.86 | 38.78 | 94.42 |
| Lcy10g009270.1 | 1063 | 122.13 | 6.65 | 44.58 | 105.97 |
| Lcy06g017110.1 | 851 | 98.57 | 6.58 | 41.42 | 92.82 |
| Lcy12g002600.1 | 1505 | 172.28 | 6.3 | 45.48 | 96.88 |
| Lcy08g011450.1 | 1156 | 130.27 | 6.81 | 46.26 | 98.23 |
| Lcy05g009640.1 | 901 | 103.54 | 8.97 | 41.72 | 100.93 |
| Lcy08g014090.1 | 1335 | 153.34 | 6.3 | 43.39 | 108.02 |
| Lcy12g002640.1 | 515 | 59.44 | 7.26 | 40.79 | 99.84 |
| Lcy06g015900.1 | 421 | 48.17 | 6.29 | 40.2 | 93.52 |
| Lcy08g015750.1 | 453 | 51.61 | 5.24 | 38.32 | 108.87 |
| Lcy07g006930.1 | 1065 | 123.41 | 6.18 | 43.64 | 104.56 |
| Lcy10g009230.1 | 1153 | 131.60 | 6.34 | 34.4 | 104.87 |
| Lcy04g000450.1 | 166 | 19.03 | 6.76 | 43.36 | 105.72 |
| Lcy10g009460.1 | 1012 | 114.73 | 5.97 | 43.04 | 109.48 |
| Lcy06g015870.1 | 733 | 83.76 | 7.49 | 34.28 | 110.44 |
| Lcy03g003500.1 | 782 | 89.91 | 5.94 | 39.44 | 98.96 |
| Lcy06g016020.1 | 727 | 84.19 | 6.45 | 42.45 | 95.68 |
| Lcy06g017090.1 | 800 | 92.99 | 7.28 | 40.29 | 93.11 |
| Lcy10g009300.1 | 1020 | 115.42 | 6.03 | 39.19 | 95.14 |
| Lcy12g002930.1 | 722 | 82.61 | 6.83 | 41.31 | 101.73 |
| Lcy08g015460.1 | 636 | 72.49 | 8.74 | 40.25 | 94.07 |
| Lcy03g000520.1 | 757 | 87.05 | 5.77 | 43.35 | 101.14 |
| Lcy03g005320.1 | 877 | 101.48 | 7.84 | 49.15 | 85.04 |
| Lcy13g013600.1 | 640 | 73.59 | 6.6 | 37.8 | 98.64 |
| Lcy04g005020.1 | 1122 | 128.83 | 5.95 | 43.49 | 104.39 |
| Lcy12g003010.1 | 1583 | 180.32 | 6.14 | 49.21 | 98.7 |
| Lcy07g007370.1 | 560 | 65.01 | 6.07 | 47.58 | 96.91 |
| Lcy04g001700.1 | 1105 | 127.69 | 7.32 | 46.17 | 102.83 |
| Lcy10g003130.1 | 372 | 42.52 | 6.84 | 50.23 | 88.04 |
| Lcy12g002820.1 | 688 | 78.20 | 5.77 | 46.04 | 99.87 |
| Lcy03g001790.1 | 451 | 52.28 | 5.48 | 39.07 | 98.31 |
| Lcy07g006900.1 | 338 | 39.10 | 5.75 | 46.57 | 106.92 |
| Lcy08g015530.1 | 1206 | 137.01 | 6.04 | 41.65 | 102.94 |
| Lcy10g009260.1 | 334 | 38.13 | 5.37 | 33.42 | 106.77 |
| Lcy07g009760.1 | 682 | 77.76 | 8.36 | 46.54 | 100.28 |
| Lcy13g013850.1 | 592 | 68.48 | 7.58 | 49.63 | 102.55 |
| Lcy02g011350.1 | 1145 | 130.54 | 7.99 | 39.16 | 107.14 |
| Lcy06g002710.1 | 1006 | 114.94 | 7.66 | 46.48 | 101.52 |
| Lcy08g010110.1 | 938 | 107.62 | 6.27 | 45.61 | 108.54 |
| Lcy08g011200.1 | 1062 | 122.38 | 6.07 | 49.69 | 108.42 |
| Lcy12g002500.1 | 1139 | 130.45 | 9.08 | 42.07 | 101.31 |
| Lcy08g011170.1 | 1175 | 134.19 | 6.69 | 44.54 | 103.34 |
| Lcy07g006870.1 | 914 | 105.52 | 7.02 | 38.55 | 98.99 |
| Lcy08g015680.1 | 1399 | 160.19 | 5.58 | 41.93 | 104.26 |
| Lcy10g010070.1 | 1163 | 133.13 | 6.75 | 40.81 | 103.07 |
| Lcy12g002920.1 | 1579 | 180.83 | 6.53 | 43.87 | 97.63 |
| Lcy02g008670.1 | 1104 | 127.54 | 8.1 | 44.81 | 101.09 |
| Lcy12g002890.1 | 1607 | 185.75 | 6.5 | 47.04 | 96.75 |
| Lcy12g020170.1 | 820 | 93.38 | 6 | 48.21 | 102.34 |
| Lcy08g005900.1 | 1291 | 148.82 | 5.91 | 47.32 | 95.63 |
| Lcy12g003040.1 | 701 | 80.15 | 5.95 | 43.96 | 94.09 |
| Lcy10g009290.1 | 440 | 49.91 | 8.04 | 30.44 | 108.05 |
| Lcy02g011380.1 | 312 | 35.10 | 8.7 | 30.25 | 99.04 |
| Lcy12g002850.1 | 989 | 111.45 | 7.01 | 39.92 | 103.67 |
| Lcy09g016520.1 | 1130 | 128.47 | 6.22 | 38 | 96.15 |
| Lcy12g002620.1 | 1664 | 189.72 | 6.52 | 46.93 | 96.51 |
| Lcy08g015540.1 | 467 | 52.78 | 5.63 | 33.8 | 98.5 |
| Lcy10g009220.1 | 1095 | 125.54 | 6.81 | 35.26 | 104.73 |
| Lcy08g015510.1 | 677 | 77.31 | 6.25 | 41.22 | 101.49 |
| Lcy10g006010.1 | 632 | 73.39 | 7.81 | 41.3 | 99.62 |
| Lcy08g011210.1 | 1180 | 136.09 | 6.15 | 44.44 | 102.53 |
| Lcy12g003070.1 | 1570 | 179.31 | 5.8 | 46.69 | 94.09 |
| Lcy08g015440.1 | 881 | 100.86 | 7.79 | 37.55 | 90.23 |
| Lcy13g012680.1 | 1108 | 126.84 | 5.97 | 42.46 | 91.6 |
| Lcy08g011140.1 | 1352 | 153.02 | 6.22 | 42.01 | 98.2 |
| Lcy07g007070.1 | 1110 | 127.50 | 5.43 | 46.17 | 102.45 |
| Lcy10g009320.1 | 560 | 64.32 | 6.04 | 41.02 | 98.82 |
| Lcy10g009340.1 | 936 | 107.37 | 7.08 | 38.62 | 102.14 |
| Lcy08g005890.1 | 973 | 112.48 | 8.11 | 44.58 | 104.3 |
| Lcy07g007350.1 | 707 | 80.74 | 6.7 | 41.48 | 96.76 |
| Lcy08g015900.1 | 817 | 93.46 | 6.54 | 38.47 | 101.65 |
| Lcy08g015760.1 | 266 | 29.87 | 8.73 | 30.75 | 104.29 |
| Lcy05g009610.1 | 905 | 104.07 | 8.7 | 41.22 | 99.28 |
| Lcy10g009240.1 | 650 | 74.52 | 7.27 | 44.01 | 105.78 |
| Lcy06g002730.1 | 1014 | 115.56 | 8.2 | 48.4 | 99.08 |
| Lcy10g004610.1 | 1038 | 119.09 | 6.22 | 39.22 | 103.08 |
| Lcy12g000860.1 | 1049 | 119.72 | 6.71 | 41.82 | 94.57 |
| Lcy11g016670.1 | 986 | 109.96 | 8.05 | 49.89 | 84.09 |
| Lcy10g006560.1 | 1128 | 131.25 | 6.77 | 49.04 | 99.31 |
| Lcy04g016910.1 | 1018 | 114.54 | 5.58 | 42.41 | 95.54 |

**Table S2. Estimates of synonymous (Ks) and non-synonymous (Ka) substitution rates and Ka/Ks ratios for two NBS-LRR paralogous pairs in *L. cylindrica*.**

| **Paralogous gene pairs** | **S-sites** | **N-sites** | **Ka** | **Ks** | **Ka/Ks** | **Selection pressure** |
| --- | --- | --- | --- | --- | --- | --- |
| Lcy06g015900.1-Lcy02g011380.1 | 210.00 | 726.00 | 0.02 | 0.03 | 0.76 | Purifying selection |
| Lcy06g015870.1-Lcy02g011350.1 | 473.92 | 1671.08 | 0.26 | 0.38 | 0.68 | Purifying selection |

**Table S3. Primers used for RT-qPCR**

| **Name** | **Forward Primer (5’-3’)** | **ReversePrimer (5’-3’)** | **ProductSize(bp)** |
| --- | --- | --- | --- |
| *Lcy04g014350* | CCTTCATTGACATTTGCACGGT | AGTCGTCATCAACTGCACACT | 129 |
| *Lcy06g017090* | CAAGCTGAGAGCTCGGTAGG | CATTTTTCGGCTAGGGATGCG | 104 |
| *Lcy08g015680* | ACAGGATTGGAGTGCATGGG | GCTCTATTGTCTCACGGGCA | 129 |
| *Lcy08g015900* | CGAGCTCTTCACCCGGAATC | TGGGCTGCTGCTCATTAGTC | 143 |
| *Lcy10g009220* | TTGAGTGTGGATCGGCTTCC | TCGTTGCTTCCCTCAAACGG | 149 |
| *Lcy10g009300* | GTCCACTGGGGCTACAACTT | TTCAGTACTCTGGGGAGGCA | 146 |
| *Lcy10g009460* | TTTTTGCAGGAAGTGCAGCC | AGACGTAAGGTTTCCCAACCA | 128 |
| *Lcy10g010070* | ACTAGGAGTGAGGAGGCTGG | TGCAGAAGCTCTTCCCTTGA | 108 |
| *Lcy12g002620* | GGTGAGCTATGAGCCTCGTG | ACGTGCACCACAGAATGCTA | 115 |
| Lc18s rRNA | GTGTTCTTCGGAATGACTGG | ATCGTTTACGGCATGGACTA | 271 |
